# From beeps to streets: unveiling sensory input and relevance across auditory contexts

**DOI:** 10.3389/fnrgo.2025.1571356

**Published:** 2025-04-28

**Authors:** Silvia Korte, Manuela Jaeger, Marc Rosenkranz, Martin G. Bleichner

**Affiliations:** ^1^Neurophysiology of Everyday Life Group, Department of Psychology, University of Oldenburg, Oldenburg, Germany; ^2^Research Center for Neurosensory Science, University of Oldenburg, Oldenburg, Germany

**Keywords:** EEG, attention, perception, real-world, auditory, ERP, everyday life, long-term

## Abstract

**Introduction:**

This study investigates the neural basis of sound perception in everyday life using EEG data recorded in an office-like environment over 3.5 h. We aimed to understand how contextual factors such as personal relevance, task complexity, and stimulus properties influence auditory processing in ecologically valid settings.

**Methods:**

By systematically increasing the complexity of acoustic scenes and tasks, we analyzed changes in neural responses, focusing on the N100 and P300 components.

**Results:**

Our results show that while the P300 is a stable marker of attention in both isolated sounds and complex soundscapes, the N100 is more sensitive to task complexity and environmental factors.

**Discussion:**

These findings highlight the importance of context in shaping auditory perception and suggest that laboratory-based findings can be partially generalized to real-world settings. At the same time, task demands significantly influence neural markers. This opens new opportunities to study sound perception in naturalistic environments without sacrificing the control typically afforded by laboratory studies.

## 1 Introduction

Complaints about noise pollution have been around for as long as people have lived together in cities. Its impact on human health and well-being is an increasingly recognized and discussed social problem that has been investigated and proven in a variety of studies (World Health Organization, 2011). It has been shown that habituation to noise varies greatly between individuals and is rarely complete (Basner et al., 2011). Heinonen-Guzejev (2008) estimates that about 20 to 43% of the general population suffers from heightened sensitivity to noise, placing them at a higher risk for negative health outcomes from noise exposure. Studies investigating the adverse effects of noise (defined as unwanted sound) on human health generally refer to the physical properties of noise, which are measurable as loudness, frequency, or sound pressure level (Basner et al., 2014). While much of the research has focused on loud noise and its direct effects, such as hearing loss, non-auditory health impacts can also result from “quiet noise”—sounds that, while not physically loud, are perceived as annoying and stressful over time (Peris et al., 2020; Mehrotra et al., 2024). This form of noise stress has been linked to cognitive deficits, sleep disturbances, depression, and increased suicide rates (Basner et al., 2011; Hygge et al., 2002; Yoon et al., 2014).

While the physical impacts of noise on health are well-documented, the question of what constitutes noise is not only highly individual, but also highly complex. While certain physical characteristics of sound, such as high volume or specific frequencies, are generally perceived as disturbing (Rimskaya-Korsakova et al., 2022), these factors alone cannot fully explain why a sound is considered noise by an individual. In everyday life, we are surrounded by a constantly changing soundscape, and the significance we attribute to these sounds varies depending on the context. Sounds that are relevant to us in one situation may be meaningless or even distracting in another. While the negative health effects of loud noise are well documented, understanding the subjective perception of noise is a significant challenge, that cannot be sufficiently explained by description of the physical properties of sound (Rimskaya-Korsakova et al., 2022).

The complexity of sound perception highlights the need to look beyond physical properties and consider contextual and intrapersonal factors (Hong and Jeon, 2015; Yong Jeon et al., 2011). Emotional state, personal relevance, current task, activity level and intentions all influence how sound is perceived and evaluated (Shinn-Cunningham and Best, 2008; Asutay and Västfjäll, 2012; Debnath and Wetzel, 2022; Rosenkranz et al., 2023; Siegel and Stefanucci, 2011; Schlossmacher et al., 2021). Sounds deemed personally relevant, such as one's own ringtone or name, command more attention than irrelevant sounds, independent of their physical characteristics (Polich, 2007; Roye et al., 2013; Holtze et al., 2021). Moreover, the context in which sound is perceived influences how it is processed (Debnath and Wetzel, 2022). In our study, we therefore pose the question of how personal relevance of a soundscape influences individual sound perception.

One commonly used approach in studying the perception of noise is based on surveys. Although qualitative surveys provide valuable insights into sound perception by accounting for contextual factors, they are prone to cognitive biases, such as priming, false memory, or the peak-end effect (Hjortskov, 2017; O'Connell and Greene, 2017; Fredrickson and Kahneman, 1993). While various studies have attempted to quantify noise perception over time and across environments (see Kjellberg et al., 1996; Paunović et al., 2009; Yoon et al., 2014), no single model has successfully captured the full complexity of what determines sound to be perceived as noise (Pierrette et al., 2012) with the subsequent potential of negative health outcomes. Multiple factors explain variance in noise ratings, including demographic factors such as gender, age and education level, as well as contextual characteristics like perceived acceptability of the noise source, noise expectation and visibility of the noise source (for a detailed review, see Pierrette et al., 2012).

However, surveys offer limited insight into how individuals perceive their current soundscape, as they have to be administered retrospectively. Even with ambulatory assessment methods, where surveys can be done in real time (i.e., as close as possible to a sound event), the challenge remains, that the process of asking a person to reflect on a sound event, may already alter their perception of it and is related to several further challenges (see Schinkel-Bielefeld et al., 2024 for a recent review).

Given the limitations of subjective survey methods, Electroencephalography (EEG) offers an objective alternative for studying how individuals process and perceive sound in real time. EEG can provide insights into the perception of various sound properties such as sound intensity as well as related cognitive processes like attentional arousal, and the detection of auditory expectation violations (e.g., Polich, 2007; Näätänen et al., 2011). Hence, EEG is a valuable tool for capturing sound perception, particularly in situations where immediate, unbiased responses are critical. Its usability in workplace settings is significant, especially in roles requiring sustained attention to a primary task, such as in aviation (Dehais et al., 2019), public transportation (Sonnleitner et al., 2014), or healthcare (Rosenkranz et al., 2023), where self-reporting is either impractical or may compromise safety.

EEG studies of sound perception have typically focused on the brain's response to isolated auditory stimuli over short periods, conducted in controlled laboratory settings that allow for only limited behavioral variation (Maselli et al., 2023; Nastase et al., 2020; Lorenzi et al., 2023). While these experiments provide valuable insights into basic auditory processing, such as differences in P200 amplitude between noise-sensitive and non-noise-sensitive individuals when exposed to noise (Shepherd et al., 2019) or how salient events suppress neural responses to ambient sound (Huang and Elhilali, 2020), they may not fully capture the complexity of sound perception in real-world situations. Although there is increasing interest in studying more complex auditory stimuli (e.g., Rosenkranz et al., 2023; Holtze et al., 2021; Jaeger et al., 2020; Schlossmacher et al., 2021; Ding and Simon, 2012; Horton et al., 2014; Mirkovic et al., 2015; O'Sullivan et al., 2015; Fuglsang et al., 2017), these controlled experiments still differ significantly from real-world experiences, where individuals encounter a rich variety of sounds, constant environmental changes, and prolonged exposure to auditory stimuli throughout the day (cf. Hasson and Honey, 2012). This raises the question of whether neural processing changes in response to naturally occurring stimuli, which might be perceived as disturbing regardless of their physical properties. The current study, therefore, aims to explore a further question: how does the neural response to auditory stimuli differ between isolated sounds and sounds embedded within a complex soundscape?

EEG studies beyond the lab are becoming increasingly popular and open up a novel approach to study the human brain and behavior (e.g., Ladouce et al., 2019; Jacobsen et al., 2021; Gramann et al., 2014; Reiser et al., 2021; Scanlon et al., 2022; Zink et al., 2016). However, only few studies have ventured beyond the lab to investigate sound processing in naturalistic environments with even fewer studies focusing on naturally occurring sounds. Straetmans et al. (2021) and Rosenkranz et al. (2023) demonstrated that responses to continuous stimuli can be recorded while participants were engaging in a bodily active task and Hölle and Bleichner (2023) and Hölle et al. (2021) showed that smartphone-based ear EEG can measure sound processing over longer periods of more than 4 h. However, beyond-the-lab studies face several challenges and the complexity of the ever-changing real world cannot be captured by EEG recordings alone but require environmental context information to account for sources of variance that are not neurally driven (Krugliak and Clarke, 2022). Also, the neural data itself leads to further challenges, such as movement artifacts (Jacobsen et al., 2021; Gramann et al., 2014) and difficulties in interpreting EEG data due to the presence of unknown artifacts (Mathias and Bensalem-Owen, 2019). Additionally, these studies must contend with lower signal-to-noise ratios, and greater environmental variability, which complicates experimental design (Ladouce et al., 2019).

While both traditional lab-based studies and beyond-the-lab studies provide critical insights, each comes with its limitations. Laboratory studies offer the advantage of precise control and measurement but lack ecological validity. In contrast, beyond-the-lab studies capture the complexity of real-world environments, but they face challenges such as movement artifacts and reduced experimental control, complicating the interpretation of EEG data.

Our study seeks to bridge these two approaches by adopting a middle stance. While remaining in a lab-based setting, we simulate a more naturalistic environment, allowing participants greater freedom in a realistic office-work-like scenario. By interacting with complex soundscapes and performing tasks with fewer constraints, participants experience a more flexible and dynamic environment, while we retain enough control to maintain experimental rigor.

Positioned between these two extremes, our study explores whether neural patterns observed in controlled lab conditions can be generalized to more naturalistic yet semi-structured environments. This balanced approach allows us to contribute to a more comprehensive understanding of auditory processing, integrating the strengths of both controlled and real-world research.

In our study, we aim to address the various questions we have raised, which are:

- How does the manipulation of personal relevance change the perception of a soundscape (in the long term)?
- How does the neural response to auditory stimuli differ between isolated sounds and sounds embedded in a complex soundscape?
- Can we monitor changes in sound perception in a realistic (office) working condition?

Specifically, we seek to deepen our understanding of how complex acoustic scenes are perceived and interpreted over extended periods. We use EEG to record participants over several hours while they are exposed to soundscapes and tasks of varying complexity. Additionally, we manipulate the behavioral relevance of the soundscape. Our goal is to enhance the interpretation of sound processing in real-world environments, contributing to a more comprehensive understanding of how sound perception is influenced by context, personal relevance, and stimulus complexity. We focus on two well-established event-related potential (ERP) components as markers of early and late stages of auditory processing: the N100 and the P300. The N100 component, typically emerging around 100 ms post-stimulus, reflects the detection of auditory events and is known to be sensitive to both the physical properties of sounds and the allocation of attention (Näätänen and Picton, 1987; Näätänen et al., 2011). In contrast, the P300 component is considered a robust indicator of cognitive processes related to stimulus evaluation and attentional relevance (Polich, 2007). Both components have been widely used in EEG studies investigating auditory attention in controlled lab settings, and more recently, in naturalistic contexts as well (Rosenkranz et al., 2023; Straetmans et al., 2021). Our selection of these components is motivated by their reliability, interpretability, and suitability for tracking both bottom-up and top-down influences on sound perception across varying degrees of ecological complexity.

## 2 Methods

### 2.1 Participants

The sample consisted of 23 participants (13 female, 10 male) aged between 21 and 37 years (mean: 25.57, standard deviation [SD]: 3.48). All participants were right-handed, had normal (or corrected-to-normal) vision and had no history of neurological, psychiatric or psychological disorders. To ensure that we only include participants with normal hearing, all participants underwent a hearing screening on a day prior to, but close to, the experiment. Audiometric threshold of at least 20 dB HL were confirmed by pure-tone audiometry at octave frequencies from 250 Hz to 8 kHz Using a SIEMENS Unity 2 Audiometer in a sound-proof cabin and with overear headphones. We screened a total of 30 participants of which 23 were eligible.

Prior to EEG recording, participants filled out the Weinstein noise sensitivity scale. The average score was at 3.366 (SD = 0.517), whereas the norm score for this inventory is 3.037 (SD = 0.572). The inventory revealed that six individuals exhibited values of at least one standard deviation above the norm score, indicating heightened sensitivity to noise. Conversely, one individual demonstrated a value below one standard deviation of the norm score, suggesting a diminished propensity for noise sensitivity relative to the average.

Participants gave written informed consent prior to the study and received monetary compensation. The study was approved by the Ethics Committee of the University of Oldenburg.

### 2.2 Procedure

The study examined the neural response to sounds, as measured by EEG. The entire study lasted approximately 6 hours (including breaks, preparation, briefing, and debriefing) to provide insight into changes in sound processing over a longer period of time. On the day of the EEG recording, participants were asked to come in with washed hair, to improve data quality. Upon arrival, the participant was taken to the laboratory and any questions about the experimental procedure were answered by the experimenter. We then asked the participant to complete two questionnaires regarding noise sensitivity and general state (described below). After that, we placed the EEG cap. After a short calibration block, we started the experiment, which consisted of six consecutive blocks of 15 to 45 min, resulting in a total recording time of 3.5 h. After each block, the participant could take a break of a self-chosen duration and received instructions for the subsequent block.

#### 2.2.1 Questionnaires: WNSS and general state

Prior to EEG data acquisition participants completed the Weinstein Noise Sensitivity Scale (Weinstein, 1978), a 21-item inventory that asks participants to indicate, on a 6-point Likert scale ranging from “strongly disagree” to “strongly agree” how much they agree with statements related to noise (such as “I wouldn't mind living on a noisy street if the apartment I had was nice.”). In addition, they completed a questionnaire about their general current state (e.g., hours of sleep, last meal, medications etc.) to ensure eligibility for the study.

### 2.3 Paradigm

All parts of the paradigm (except for the transcription task) were presented using the Psychophysics Toolbox extension (Brainard 1997; Pelli 1997; Kleiner et al. 2007, version: 3) on MATLAB 2021b.

#### 2.3.1 General structure of block sequence

There were six consecutive blocks, in which the sounds and an additional task became progressively more naturalistic and complex. The design of the blocks was chosen so that results could be compared between them, since only one aspect changed between two consecutive blocks: auditory stimuli, non-auditory task, or listening mode.

#### 2.3.2 Experimental phases

The overall idea was to obtain measures of neural sound processing in three phases and under conditions of varying naturalness. The first phase involved passive listening, the second phase focused on active listening, and the final phase returned to passive listening.

First, we were interested in how task irrelevant background sounds are processed. For this we measured brain activity free of any auditory task to artificial and natural sounds and under varying naturalness of the additional task. In this phase, participants were instructed that the soundscape was not particularly relevant and that they could ignore it. This allowed us to compare neural processing under different levels of stimulus naturalness and the influence of task context.

Second, we manipulated participants' focus on the sounds by up-modulating the relevance of specific sounds, i.e., a change in pitch in blocks with isolated stimuli and the occurrence of a church bell in blocks with ambient sound. In this phase, participants were asked to respond to these sounds by pressing a key on the keyboard, which required active listening. This allowed us to compare the neural response (quantified as the amplitude of the N100 and P300 component of the ERP) of passive vs. active listening for the different auditory materials and the non-auditory tasks.

**Figure 1:**
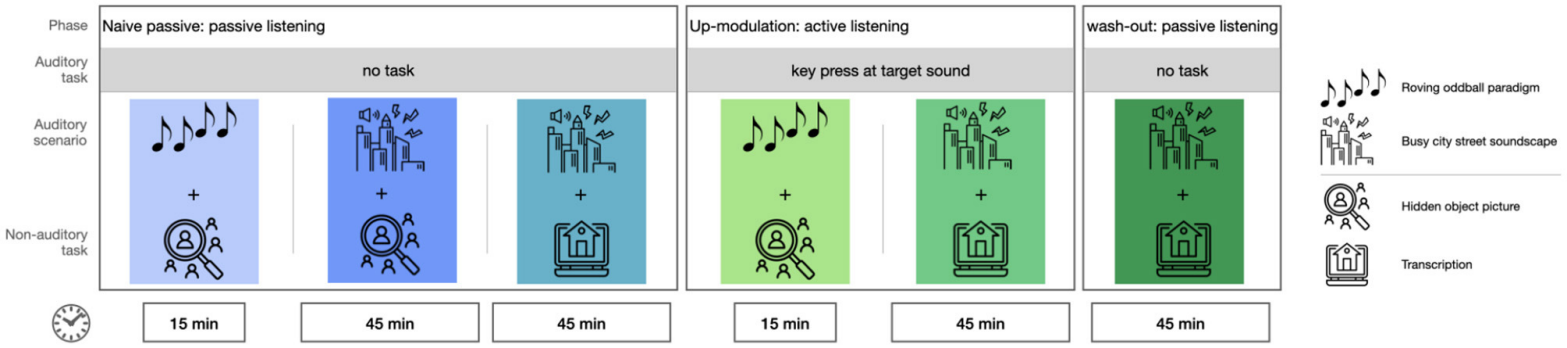
Overview of experimental blocks. Order of the blocks is chosen to ensure naivety concerning target sounds in the naive passive conditions.

Third, we asked participants to ignore the previously relevant auditory features again to obtain a measure of what we call “attentional wash-out.” Our prediction was that participants' P300 response would take some time to return to their baseline level. Thus, we predicted that attention would be washed out over time. For a visual representation of the study design, see Figure 1.

#### 2.3.3 Auditory stimuli

All sound files had a sampling rate of 44.1 kHz. Two auditory scenarios were presented. The first scenario was a roving oddball paradigm (Garrido et al. 2008), based on code publicly available on github.com (Sumner, 2022). In this paradigm 7 stereo pure tone beeps that differed in frequency (500 Hz, 300 Hz, 250 Hz, 200 Hz, 150 Hz, 100 Hz, 50 Hz) were used, with a length of 70 ms and a rise and fall time of 10 ms. For the roving oddball, sequences (trains) of these tones were generated. For each train, a tone at a random pitch was chosen and repeated 5 times, followed by a random addition of 1 to 5 more of the same tone. This resulted in trains ranging from 6 to 10 tones in length. The inter-stimulus interval between tones was 1,000 ms. No subsequent train contained the same tones as the preceding train. The roving oddball had a total duration of 15 mins, resulting in an average of 121 trains. The average volume of the tones was at 55 dB(A).

The second scenario was a pre-recorded soundscape of a busy city street, available on Youtube.^1^ It consisted of a variety of ambient sounds, such as streetcars, motorcycles or incomprehensible speech. The street scenario had a total length of 2 h and 21 min, from which we took four segments of 45 min each. These segments had a short overlap, since the original sound file was not long enough to cover three non-overlapping hours. The order in which the segments were presented was randomized across participants. Inspired by Brink et al. (2011), we added a single chime of a church bell (also taken from Youtube^2^) to create an auditory recognition task. Although the church bell sound was played at its total duration of 2,890 ms its perceptual onset and peak energy were located at the beginning of the sound. The total length was determined by its natural decay. Thus, EEG responses were effectively time-locked to the onset, comparable to shorter stimuli like the 70 ms roving oddball tones. The church bells were added at 50 random times, with an average distance of 53 seconds between bells. The bells were varied in their spatial position (i.e., they were played from the left, right, or both sides).

Accordingly, we added bird calls of three different bird species (blackbird, great tit, and sparrow) at 50 random time points and with different positions. All bird call files were modified to the same length of 790 ms to facilitate data processing later on. While the sharpness of the onset varies slightly between species, all calls exhibit prominent onsets suitable for ERP alignment. All bird calls show a typical, repetitive sound pattern composed of short, distinct vocal phrases. Each call has clear harmonic or tonal structures, with sound energy concentrated in the mid-frequency range, roughly between 2–8 kHz. While their style and complexity differ (e.g., the blackbird being more melodic), the building blocks—repetition, mid-frequency emphasis, short motifs, and tonality—are consistent. Based on these similarities, we grouped all bird calls into one sound category (Zuk et al., 2020). As the onset of a bird call represents a salient acoustic event expected to elicit a clear ERP response (Straetmans et al., 2021), we carefully time-aligned the onset of all bird calls to derive the averaged ERP response.

We embedded the church bell and bird calls at a low volume, making them less noticeable compared to the background noise. The sounds were chosen to be natural in the overall soundscape of the busy city. This approach was inspired by the study by Schlossmacher et al. (2021), where words were embedded in noise and were only recognized by the participants when they were made aware of the words. The resulting overall soundscape had an average volume of 51 dB(A).

#### 2.3.4 Non-auditory task

Participants engaged in one of two non-auditory tasks throughout the experiments depending on the block. The first task was a hidden object picture task involving visual search. A hidden object picture is a static picture with a high level of detail. There are a variety of visual objects and activities and participants had to search for a specific aspect of the picture (e.g., “Find the black cat”). The task is similar to “Where is Waldo?/Where is Wally?,” the famous children's game.

The hidden object pictures were comic-style and showed different scenes, such as a library, a playground or a hair salon. In each picture, 40 different objects were marked as targets. Participants were tasked with searching for individual objects, and the target object was written at the top of the screen throughout. Using the mouse, they could click on any object. If they clicked on the target, a message on the screen informed them of the successful identification. If they clicked any other object, they received a message instructing them to continue searching. Participants had unlimited time and unlimited attempts to find the target object. If they could not find a target or were uncertain about a word, they could skip to the next target by pressing the right arrow key on the keyboard. Participants had to search for an object for at least 30 seconds before they could choose to skip an object. If participants identified all 40 objects in a picture before the end of an experimental block, they were presented with a new hidden object picture. Participants were presented with a maximum of 5 hidden object pictures (depending on their individual task speed). The number of targets was deliberately set so high that participants would not be able to find all the targets in the allotted time, to ensure that all blocks were of equal length for all participants. The order of the pictures was randomized across participants.

The second task was a transcription task that resembled simple office work. The task was taken from the citizen science project “world architecture unlocked” on “Zooniverse” and consisted of transcribing handwritten information about architectural photography^3^. The handwritten information had to be matched to given categories, such as city, name of architect, or name of building. The task required no prior knowledge about architecture, yet was challenging enough that participants had to stay focused to complete the task. Participants were encouraged to use online search engines and online maps to correctly match the handwritten information under the photos to the required categories. The combination of deciphering, typing, and searching the internet met the requirement of resembling office work and provided higher task complexity than the hidden-object picture task.

#### 2.3.5 Experimental blocks

The experimental phases described above were divided into 6 consecutive blocks, as can be seen in Figure 1. The naive passive phase had 3 blocks, the up-modulation phase had 2 blocks and the wash-out phase had one block.

Both auditory scenarios were presented in either passive or active listening mode. In the passive listening mode, participants were informed that there would be background noise but that it was irrelevant, and could be ignored. In the active listening mode, participants were asked to respond to specific acoustic events for each of the auditory scenarios. For the roving oddball, they were asked to indicate a change in pitch from one tone to the next by pressing the F4 key on the keyboard. For the street scenario, they were asked to indicate the occurrence of the church bell by pressing the F4 key. The response key was chosen to avoid a potential conflict in the non-auditory task, where participants also had to use the keyboard. All other sounds, including the bird calls, were not behaviorally relevant and never required a response. They were used to facilitate the ERP analysis, so that the relevant events (i.e., church bells) could be compared with non-relevant events (i.e., bird calls).

In detail, the experimental blocks were as follows:

- Block 1 (Naive passive listening): Participants listened to a 15-minute sequence of the roving oddball while completing the hidden object picture task. They were not required to respond to the sounds.
- Block 2 (Naive passive listening): Participants listened to a 45-minute sequence of the street soundscape while working on the hidden object picture task. They were not required to respond to the sounds.
- Block 3 (Naive passive, passive listening): Participants listened to a 45-minute sequence of the street soundscape while performing the transcription task. They were not required to respond to the sounds.
- Block 4 (Active listening, up-modulation): Identical to Block 1, but now participants were instructed to respond to the first tone of each train.
- Block 5 (Active listening, up-modulation): Identical to Block 3, but now participants were instructed to respond to the church bell sounds.
- Block 6 (Passive Listening, wash-out): Identical to Block 3. Mind that participants were now required to ignore the previously relevant church bell sound again. They were not required to respond to the sounds.

The order of blocks was identical for all participants. This was necessary to assure that participants were naive to the target sounds in the passive listening blocks.

### 2.4 Hypotheses

We analyzed the EEG data for early and late components of the ERP which are the N100 component, known to reflect early auditory processing and stimulus intensity (Näätänen and Picton, 1987) and the P300 component, known to reflect higher cognitive processing, such as attention (Polich, 2007). We postulated the following hypotheses.

#### 2.4.1 Attention modulation of target processing

Hypothesis 1: The amplitude of the P300 component to target stimuli will be greater for a target sound in the active listening condition compared to the passive listening condition. This effect has been consistently found in laboratory experiments (e.g., Polich, 2007). To test this hypothesis, we compared the ERPs from block 1 (passive listening to the roving oddball + hidden object picture task) to block 4 (active listening to the roving oddball + hidden object picture task) and from block 3 (passive listening to the street soundscape + transcription task) to block 5 (active listening to the street soundscape + transcription task).

#### 2.4.2 Attention modulation of non-target processing

Hypothesis 2: Processing of behaviorally irrelevant sounds, as represented by the N100 of the ERP to non-relevant beeps in the roving oddball and non-relevant added bird sounds in the street soundscape, will generally be larger in the active listening condition than in the passive listening condition because participants have to scan the entire soundscape for the target. To test this, we compared the same blocks as in hypothesis 1.

#### 2.4.3 Contextual influence on the P300 component

Hypothesis 3: We hypothesized a difference in P300 amplitude for relevant features of the auditory scene in active vs. passive listening between the blocks with more controlled experimental features (i.e., roving oddball + hidden object picture task) and blocks with less controlled experimental features (i.e., street soundscape + transcription task). We predicted that the P300 amplitude would show a higher amplitude in active vs. passive listening in blocks with more experimental control than in the blocks with less experimental control. To test this, we compared the grand average between the effects of block 1 (hidden object picture task + passive roving oddball) and block 4 (hidden object picture task + active roving oddball) vs. block 3 (transcription task + passive naive street soundscape) and block 5 (transcription task + active street soundscape).

#### 2.4.4 Task complexity effects on early auditory processing (N100)

Hypothesis 4: The amplitude of the N100 component of the ERP will be greater during passive listening to the street soundscape when working on the hidden-object picture task compared to the transcription task. We hypothesized that the transcription task, resembling simple office work, would elicit a smaller N100 component for relevant (bell) as well as irrelevant (bird) sounds in the street soundscape compared to the street soundscape while working on the hidden object picture task. To test this hypothesis, we compared block 2 (passive listening to the street soundscape + hidden object picture task) to block 3 (passive listening to the street soundscape + transcription task).

#### 2.4.5 Learning and unlearning of auditory relevance

Hypothesis 5: We hypothesize a gradual down-modulation of the personal relevance in the street soundscape in the last block (transcription task + wash-out street soundscape), as indicated by a reduction in P300 amplitude over time. We compared the P300 amplitudes of the data in increments of thirds between the passive naive listening condition and the wash-out condition. Our expectation was that there would be a significant difference between each first third but not between each final third.

### 2.5 Data acquisition

#### 2.5.1 Description of lab setup

Participants were seated in a soundproof recording booth at a desk with a screen (Samsung, SyncMaster P2470) in front of them. The auditory material was presented free-field through two loudspeakers (Sirocco S30, Cambridge Audio, London, United Kingdom) positioned at a 45-degree angle to the left and right and at a distance of approximately 0.5 m at ear level. A mouse and a keyboard were placed on the desk in front of the participant which were used to indicate target sounds and for working on the non-auditory tasks. For relevant events in the auditory scenes and additional tasks, a marker was generated using the Lab Streaming Layer (LSL) library.^4^ Keyboard input was recorded using key capture software from the LSL library.^5^ The Lab Recorder software^6^ was used to ensure the temporal synchronicity of the EEG data, the event markers and the keyboard capture. Files were saved as .xdf and organized using the Brain Imaging Data Structure (BIDS) format (Gorgolewski et al., 2016) with the EEG data extension (Pernet et al., 2019).

#### 2.5.2 EEG system

We used a 24-channel EEG cap (EasyCap GmbH, Hersching Germany) with passive Ag/AgCl electrodes (channel positions: Fp1, Fp2, F7, Fz, F8, FC1, FC2, C3, Cz, C4, T7, T8, CP5, CP5, CP1, CPz, CP2, CP6, TP9, TP10, P3, Pz, P4, O1, and O2). The mobile amplifier (SMARTING MOBI, mBrainTrain, Belgrade, Serbia) was attached to the back of the participants' head in a small pocket of the EEG cap, so that they could move their head freely throughout the experiment (and it was also easy to leave the lab, e.g., for a bathroom or lunch break). In addition, we collected gyroscope data from the amplifier to track the participants' head movements. The data were transmitted via Bluetooth using a Bluetooth dongle (BlueSoleil) connected to a desktop computer that was also used for stimulus presentation. EEG and gyroscope data were transmitted to LSL via the SMARTING Streamer software (v3.4.3; mBrainTrain, Belgrade, Serbia) and recorded with the lab recorder at a sampling rate of 250 Hz.

#### 2.5.3 Measurement procedure

After applying the cap, the skin beneath each electrode was cleaned with 70% alcohol and abrasive gel (Abralyt HiCl, Easycap GmbH, Germany). The electrodes were then filled with abrasive gel to improve the conductance between the skin and the electrodes. Impedances were kept below 10 kΩ at the beginning of data collection and were checked and improved as necessary between the blocks. In total, re-gelling was required for 9 out of 24 participants. For 7 of these participants, a single re-gelling step was sufficient during the experiment. One participant required two re-gelling steps, and another required three. In most cases, re-gelling concerned only one electrode. In two cases, 2 and 3 electrodes respectively had to be re-gelled. Given a total of 24 electrodes per participant, the need for re-gelling was minimal, and no full cap removal or major intervention was necessary.

Given the length of the experiment, all participants were asked to bring food to allow for an extended lunch break between blocks. The time of the lunch break was flexible, with the only restriction that the break could not be between the last two blocks (active listening and wash-out) so as not to compromise the experimental manipulation.

### 2.6 Data analysis

All data analysis was carried out using MATLAB 2021b with the EEGLAB toolbox (Delorme and Makeig, 2004; version: 2021.1).

#### 2.6.1 EEG pre-processing

To clean the EEG data, we applied a pre-processing to it. First, we filtered the data between 1 and 40 Hz (default settings of the pop_eegfiltnew function). Then we detected bad channels, using the clean_artifacts function of EEGLAB with “channel_crit_maxbad_time” at default setting and stored them for later interpolation. We then cut the data into segments of 1 second length. For these segments, we applied an artifact rejection with a probability criterion of +/– 3 SD from the mean. This was done to improve the independent component analysis (ICA) training. Second, we combined all the data obtained from the first cleaning step to compute the weights for an ICA. We used the runica function from EEGLAB and chose the extended training method. These ICA weights were then applied to the raw data of all blocks. For component rejection, we applied the ICLabel algorithm (Pion-Tonachini et al., 2019) and rejected components with a probability ≥ 80% of being an artifact of any class (i.e.,: eye, muscle, heart, other). Additionally, we rejected components based on visual inspection where necessary, as the ICLabel algorithm is only optimally trained on stationary data where participants were constrained in their movements, which does not fully match the characteristics of our setup, where participants were free to move within the scope of the task. On average, we had to remove 8 out of 24 components per participant (min = 4, max = 11) After ICA cleaning, we filtered the data (low pass: 0.5 Hz, high pass: 20 Hz), interpolated any bad channels (1.4 on average, min = 0, max = 5) and re-referenced the data to the mean of electrodes Tp9 and Tp10 (these never had to be interpolated).

#### 2.6.2 ERP analysis

Prior to epoching, we corrected audio events for a constant delay of 35 ms. EEG data were then epoched around the audio events of interest. For the roving oddball (blocks 1 and 4), we epoched between –200 and 1,000 ms relative to the onset of the first tone and last tone of each train. For the street soundscape (blocks 2,3,5, and 6), we created epochs of 2.2 seconds length (–200 to 2,000 ms) around each event of a church bell and each event of a bird call. The longer epoch duration in the street soundscape condition accounted for the longer response window, as explained above. We found a time-locked artifact in the active street condition (block 5) in the time range of reaction times that can be explained by the fact that some participants were moving their head down to face the keyboard and find the response key when a target appeared. This artifact is especially pronounced in the frontal electrodes but does not contaminate the time windows of interest for the N100 and P300 analysis.

Epochs for both conditions were baseline corrected (–200 to 0 ms) and epochs that deviated at least 3 SD from the mean were rejected. For the first tones of the roving oddball we rejected on average 9.48 trials (min = 2, max = 20), For the last tones of the roving oddball we rejected on average 10.14 trials (min = 4, max = 21). For the bell epochs in the street soundscape we rejected on average 4.78 trials (min = 1, max = 9) and for the bird epochs we rejected on average 4.93 trials (min = 1, max = 11). The remaining epochs were used for the ERP analysis. For analyzing the P300 component, we did a channel selection of channels P3, Pz, and P4, since the parietal channels usually show the highest contribution to the P300 peak of the ERP (Polich, 2007). For analyzing the N100 component we selected frontal channels FC1, Fz, and FC2 (Näätänen and Picton, 1987).

Supplementary Table 1 holds the overview of sample sizes per block after correcting for corrupted or missing data.

#### 2.6.3 Behavioral data

For the auditory task, we calculated hits and false alarms for the target sounds. Additionally, we calculated the response times for the hits to the auditory task in the active listening blocks. For the roving oddball, a hit was defined as a response key press after each first tone of a train within a time range of 200–1,000 ms after tone onset. A false alarm was defined as a response key press after any tone other than the first tone of a train. Similarly, for the street soundscape, a hit was defined as a response key press after the church bell (time range: 200–2,000 ms), while a false alarm was defined as a response key press at any other time or event. The longer response window for the street soundscape was chosen because participants could not rest their hand on the response key while working on the transcription task, as was possible (and encouraged) for the hidden object picture task. Moreover, the longer response window accounted for the increased task difficulty, introduced by the background noise, resulting in an auditory discrimination task instead of an auditory detection task as in the roving oddball condition (Deshpande et al., 2022). To assess the consistency of performance, we computed hit rates and false alarm rates and used the interquartile range (IQR) method to detect outliers. Specifically, we defined false alarm outliers as values above *Q*3 + 1.5 × *IQR*, and hit rate outliers as values below *Q*1 − 1.5 × *IQR*.

For the hidden object picture task, we looked at the average number of objects correctly identified in 15 min and examined whether task performance changed over time (i.e.: participants identified more/less objects per block). Because the transcription task was implemented using a public citizen science platform, no information was available on the correctness or total number of completed assignments. To get an estimate of the overall task engagement of the participant in this task, we calculated the number of keystrokes per minute.

#### 2.6.4 Statistical testing

To determine whether the data met the assumptions of parametric statistical tests, we conducted a Shapiro-Wilk normality test on the ERP data for each condition. The results indicated significant deviations from normality (*p* < 0.05) in several comparisons. Consequently, we used the non-parametric Wilcoxon signed-rank test for testing the hypotheses and the task performances in the additional tasks to account for these violations. We assumed evidence for an effect at α = 0.05. To control for Type I errors (false positives) arising from multiple comparisons, we applied a Bonferroni correction for multiple testing in our analysis. Given that certain data blocks (especially bell-epochs from block 3) were used across multiple hypotheses, we employed an overarching correction approach. This method ensures that all comparisons involving the same dataset are accounted for across different hypotheses, rather than treating each hypothesis in isolation. For each hypothesis, a unified correction was applied based on the maximum number of comparisons in which the same data block was used across all hypotheses. This led to a correction for 6 comparisons in hypotheses 1, 3, 4, and 5, resulting in an adjusted alpha level of 0.0085 (0.05/6) and 2 comparisons in hypothesis 2, resulting in an adjusted alpha level of 0.025 (0.05/2; detailed explanation in supplement). A result was only considered statistically significant if the *p*-value was below the respective adjusted alpha level. For comparisons of the N100 amplitude, we used the average over channels FC1, Fz, and FC2. For comparisons of the the P300 amplitude, we used the average over channels P3, Pz, and P4.

The time windows for the ERP components were determined based on visual inspection of the grand-average waveforms. We observed that the components stemming from the street soundscape conditions were delayed compared to those from the oddball paradigm. We assume that this delay is introduced by the softer volume of the added sounds, which makes their perceptual onset less distinct. Additionally, masking effects from the continuous background noise of the street soundscape may contribute to a further perceptual delay. Based on these observations, we adjusted the time windows accordingly. In blocks, where the roving oddball was applied, the time window for the N100 was set to 50–160 ms and the time window for the P300 was set to 350–800 ms. In case of the street soundscape we used a time window of 90–200 ms for the N100 analysis and a time window of 450–900 ms for the P300 analysis. Time windows were averaged across their whole length. Although the timing was adjusted to better capture the components in each condition, the duration of the time windows remained identical across conditions (550 ms). Longer overall epochs were used in the street soundscape condition for visualization purposes, in order to show broader ERP components and response-related artifacts.

## 3 Results

### 3.1 Auditory task performance

The average hit rate in the active roving oddball was at 81.56% (SD = 20.79), and the average false alarm rate was 13.25% (SD = 16.85%), based on 120 tone changes. We identified 3 outliers based on false alarm rate and 4 outliers based on hit rate. Excluding these participants, the false alarm rate dropped to 7.32% (SD = 4.64%), and the hit rate increased to 90.38 % (SD = 7.36%), indicating more homogeneous performance in the remaining sample.

**Figure 2:**
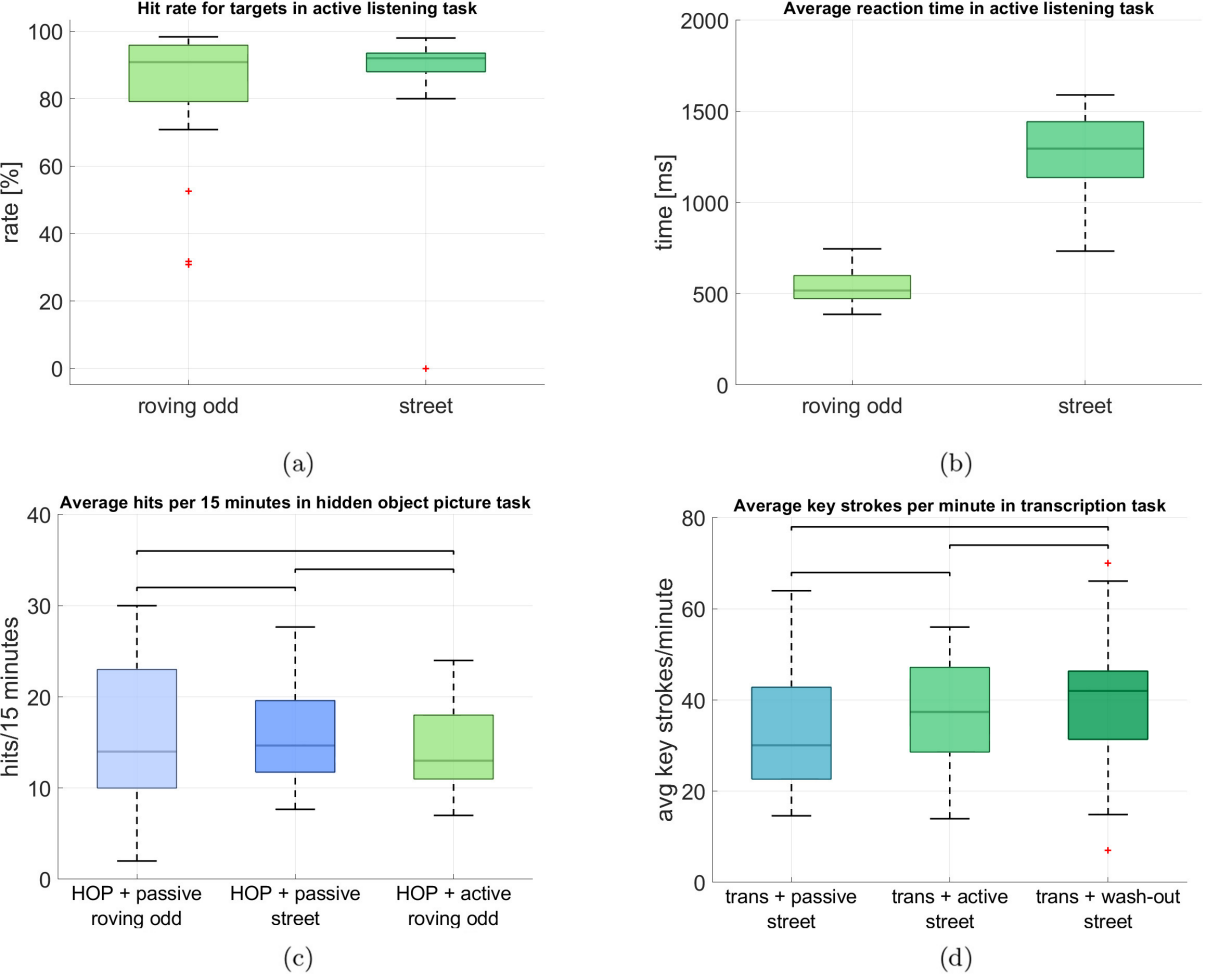
Behavioral task performances in the auditory and non-auditory tasks. The central line in each box represents the median. The lower and upper edges of the box indicate the 25th and the 75th percentiles, respectively. The whiskers extend to the most extreme data points within 1.5 × the interquartile range, while data points beyond this range are considered outliers and are displayed as individual markers. **(a)** Comparison of the hit rate in the auditory tasks in active listening. **(b)** Comparison of reaction times in the auditory tasks in active listening. **(c)** Comparison of the hit rates for the 3 blocks in which the hidden-object picture task was performed. Data is displayed as a 15-min-average to ensure comparability between blocks. **(d)** Comparison of the average absolute number of key strokes per minute in the transcription task.

The average hit rate for the active street soundscape was comparable at 86.52% (SD = 19.50) and the false alarm rate was 1.83% (SD = 2.08%), based on 50 occurrences of the church bell. No false alarm outliers were identified, while 4 participants were classified as outliers based on hit rate. After excluding these, the hit rate increased to 92.21% (SD = 3.19%), suggesting robust and consistent performance across participants in this more ecologically complex condition. Although the numbers are comparable, it can be seen in Figure 2a, that there was a higher variance in the performance in the roving oddball.

The average reaction time in the roving oddball was at 525.79 ms (SD = 82.01) and 1,258.10 ms (SD = 235.50) in the street soundscape, as can be seen in Figure 2b. The large difference can partly be explained by the fact that for the roving oddball participants could rest their left hand on the target key, such that they could immediately press it when they heard a target. In the street soundscape condition, participants were asked to press the same key but they were not able to rest their hand on it, since they needed both hands for typing during the transcription task. The roving oddball yielded a smaller variance in reaction times, while there was a larger variance in the street soundscape condition.

### 3.2 Additional task performance

Figures 2c, d show the performance in the additional tasks (i.e., hidden object picture and transcription). For the hidden object picture task (Figure 2c), we display the average number of correctly identified targets in 15 minutes to ensure comparability between the blocks, since they differed in total length (15 min vs. 45 min). Under passive naive listening of the roving oddball (block 2), participants on average correctly identified 16.35 targets (SD = 8.29). Under passive naive listening of the street soundscape they had an average performance of 15.80 targets (SD = 5.69) and in the active listening block they identified on average 14.14 targets (SD = 4.99).

To obtain a behavioral measure for the transcription task, we counted the average number of keyboard strokes per minute and participant for each block (Figure 2d). Under passive naive listening of the street soundscape (block 3), participants had on average 34.23 key strokes per minute (SD = 14.78). In the active listening condition (block 5) they had an average of 37.85 (SD = 13.09) and in the wash-out out condition (block 6) they had an average of 40.40 (SD = 15.37), It can be seen that in both tasks there is no clear pattern of performance gain or loss over time as could have been expected especially for the transcription task, where participants had to learn the task initially. We tested this using Wilcoxon signed-rank test and corrected for 3 comparisons to investigate the group differences for each block of the hidden object picture task and the transcription task separately. There were no significant differences between the blocks for the performance in the hidden object picture task (HOP + passive roving odd vs. HOP + passive street: adj. *p* = 0.945, Z = 0.069; HOP + passive roving odd vs. HOP + active roving odd: *p* = 0.088, Z = 1.707; HOP + passive street vs. HOP + active roving odd: *p* = 0.158, Z = 1.213). Also, there were no significant differences between the blocks for the transcription task (trans + passive street vs. trans + active street: *p* = 0.128, Z = –1.521; trans + passive street vs. trans + wash-out street: *p* = 0.039, Z = –2.100; trans + active street vs. trans + wash-out street: *p* = 0.189, Z = –1.315).

### 3.3 Hypotheses testing

#### 3.3.1 Attention modulation of target processing—hypothesis 1

**Figure 3:**
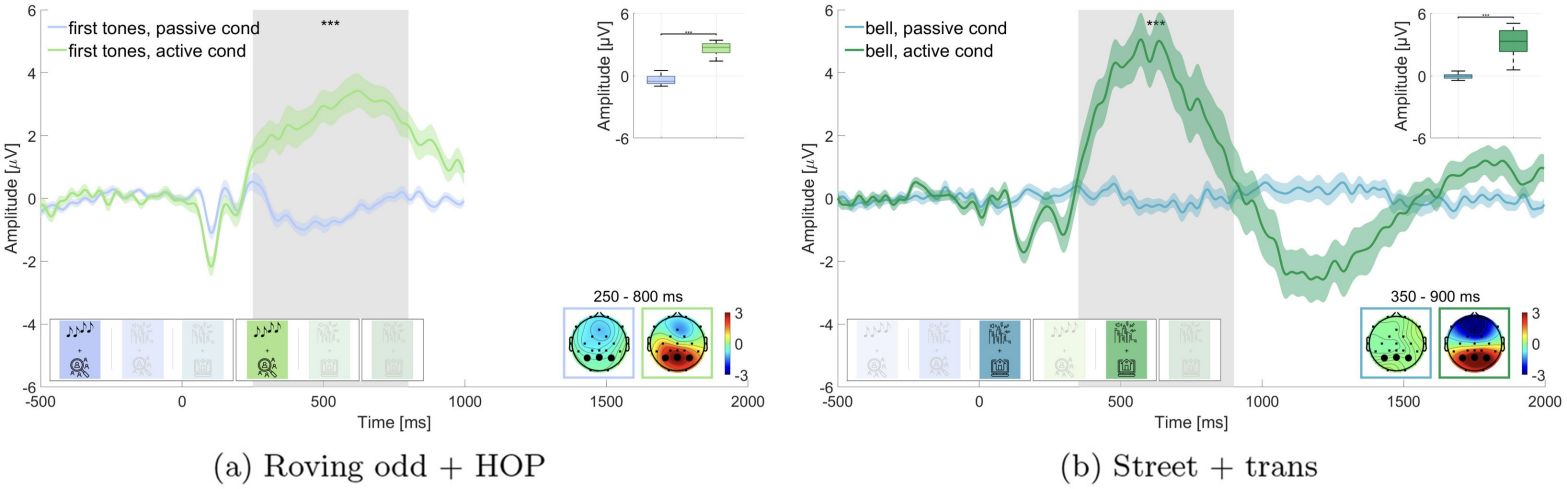
Comparison of the P300 amplitude for target sounds in passive vs. active listening. Graphs show the average over the marked channels (black circles) in the topographies. Shaded regions mark the standard error of the mean. Asterisks indicate the level of significance (^*^p ≤ 0.05; ^**^p ≤ 0.01; ^***^p ≤ 0.001). **(a)** Roving oddball (roving odd) conditions. First tones in passive vs. active listening while performing the hidden object picture task (HOP). **(b)** Street conditions. Bell sounds in passive vs. active listening while performing the transcription task (trans).

We expected a larger P300 amplitude to target stimuli (i.e., first tones in roving oddball and bell sounds in street soundscape) in the active listening condition as compared to the passive listening condition. We compared the respective blocks for the roving oddball (i.e., block 1 and 4) and the street soundscape (i.e., block 3 and 5) separately and performed a Wilcoxon signed-rank test for each comparison. We found significant differences between the active listening and the passive listening condition for the first tones in the roving oddball (adj. *p* ≤ 0.001, Z = –10.192, adjusted for 6 comparisons, Figure 3a) as well as for the bell sounds in the street soundscape (adj. *p* ≤ 0.001, Z = –10.192, adjusted for 6 comparisons, Figure 3b), as displayed in Figure 3. The mean amplitudes in the time window of interest for the roving oddball were M = –0.402 μV (SD = 0.415 μV) in the passive listening condition and M = 2.631 μV (SD = 0.542 μV) in the active listening condition. For the street soundscape, we found a mean amplitude of M = –0.035 μV (SD = 0.222 μV) in the passive listening condition and M = 3.294 μV (SD = 1.252 μV) in the active listening condition. We found evidence for the first hypothesis being in line with existing literature (e.g., Polich, 2007).

#### 3.3.2 Attention modulation of non-target processing—hypothesis 2

**Figure 4:**
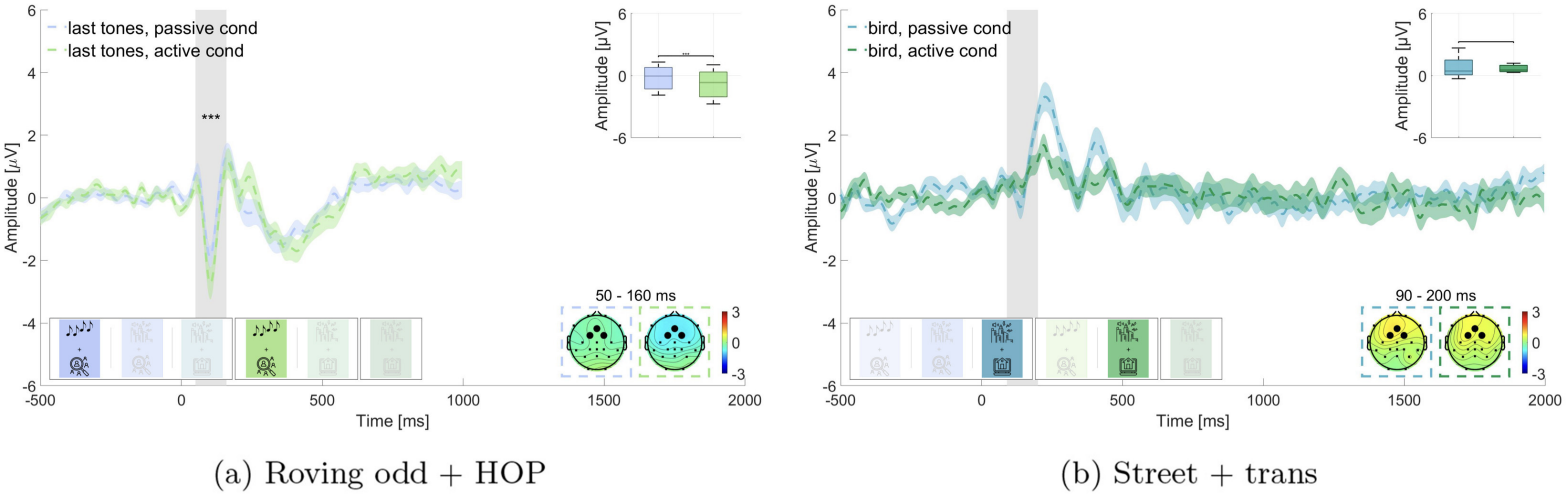
Comparison of the N100 amplitude for non-target sounds in passive vs. active listening. Graphs show the average over the marked channels (black circles) in the topographies. Shaded regions mark the standard error of the mean. Asterisks indicate the level of significance (^*^p ≤ 0.05; ^**^p ≤ 0.01; ^***^p ≤ 0.001). **(a)** Roving oddball (roving odd) conditions. Last tones in passive vs. active listening while performing the hidden object picture task (HOP). **(b)** Street conditions. Bird sounds in passive vs. active listening while performing the transcription task (trans).

We expected that irrelevant sounds in the same blocks as for hypothesis 1 (i.e., block 1 and 4: last tones and block 3 and 5: bird sounds) would elicit a larger N100 amplitude in the active listening than in the passive listening condition. Results are displayed in Figure 4. Wilcoxon signed-rank test revealed a significant difference for the roving oddball, where the amplitude of last tones was significantly larger in the active listening than in the passive listening condition (*p* ≤ 0.01, Z = 4.623, adjusted for 2 comparisons, Figure 4a). The mean amplitude in the time window of interest was M = –0.212 μV (SD = 1.100 μV) in the passive listening condition and M = –0.830 μV (SD = 1.269 μV) in the active listening condition. This was not found for the street soundscape, where bird sounds did not show a statistically significant difference between the two conditions (adj. *p* = 0.539, Z = 0.615, adjusted for 2 comparisons, Figure 4b). Here the amplitude in the time window of interest was M = 0.769 μV (SD = 0.920 μV) in the passive listening condition and M = 0.647 μV (SD = 0.298 μV) in the active listening condition. We found evidence in favor of hypothesis 2 concerning the effect in the roving oddball but not concerning the effect in the street soundscape.

#### 3.3.3 Contextual influence of the P300 component—hypothesis 3

**Figure 5:**
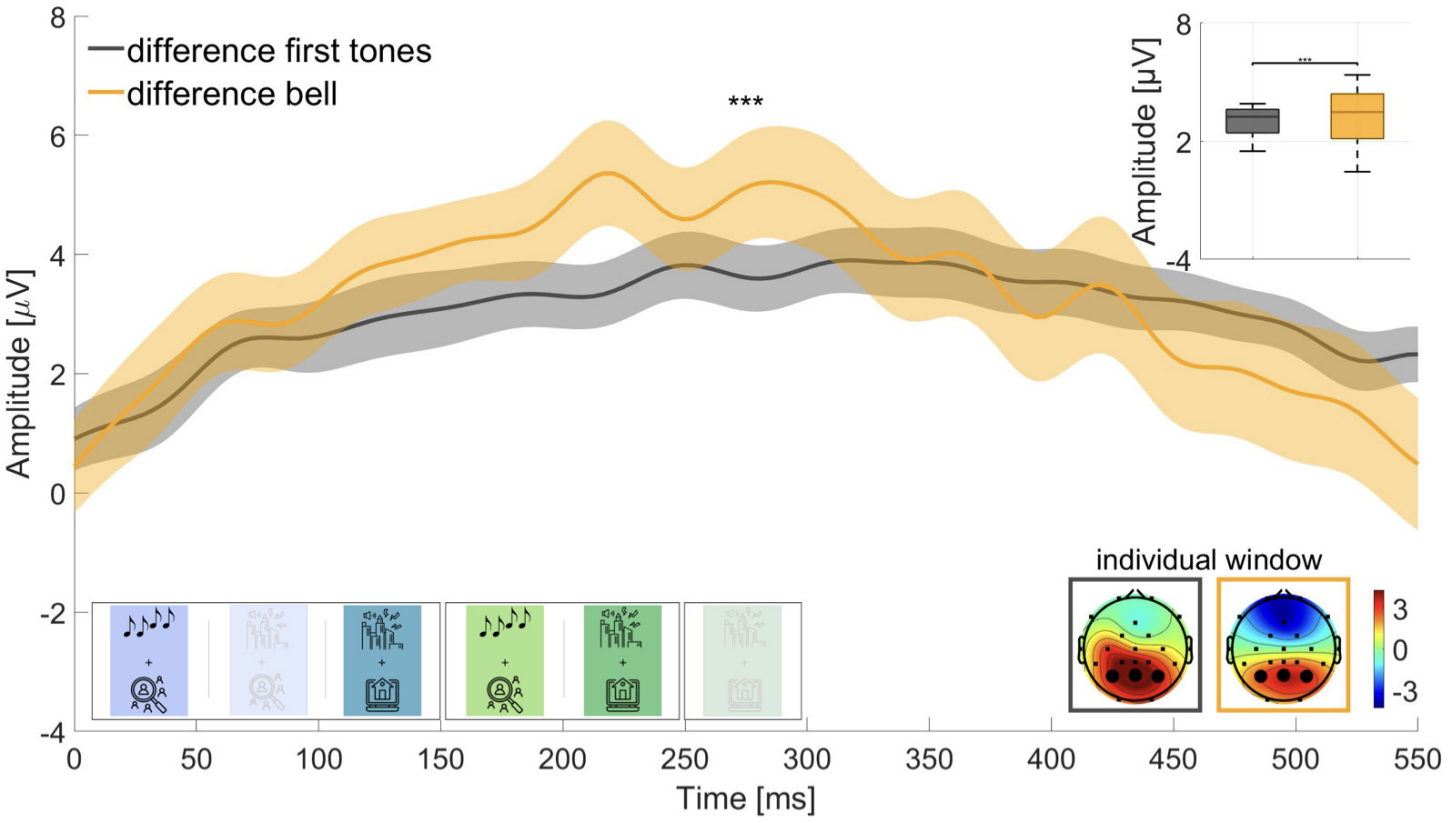
Difference waves for the P300 amplitude to target sounds in active minus passive listening. For the roving oddball: First tones in active listening minus first tones in passive listening (time window: 350–800 ms); for the street soundscape: Bell sounds in active listening minus bell sounds in passive listening (time window: 450–900 ms). Graphs show the average over the marked channels (black circles) in the topographies. Shaded regions mark the standard error of the mean. Asterisks indicate the level of significance (^*^p ≤ 0.05; ^**^p ≤ 0.01; ^***^p ≤ 0.001).

We were interested in the magnitude of the difference between P300 amplitudes in active and passive listening and expected a larger amplitude for the difference wave of the roving oddball conditions than for the street soundscape conditions. Therefore, we computed the difference waves for first tones in active listening (block 4) minus first tones in passive listening (block 1) and for bell sounds in active listening (block 5) minus bell sounds in passive listening (block 3). Wilcoxon signed-rank test revealed that the two waves were significantly different (adj. *p* ≤ 0.001, Z = –3.629, adjusted for 6 comparisons), but in the opposite direction than expected (see Figure 5). The difference wave for the street soundscape showed a larger amplitude (M = 3.329 μV, SD = 1.341 μV) than the difference wave for the roving oddball (M = 3.033 μV, SD = 0.741 μV). We therefore could not find evidence for hypothesis 3.

#### 3.3.4 Task complexity effects on early auditory processing (N100)—hypothesis 4

**Figure 6:**
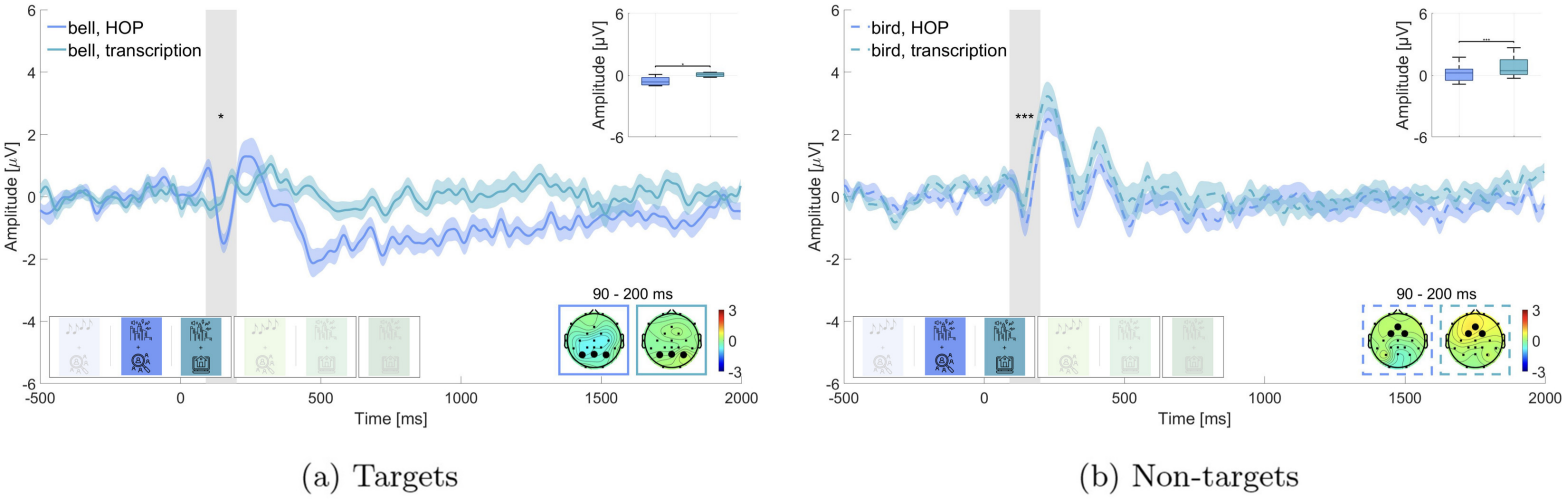
Comparison of the N100 amplitude in passive listening while either performing the hidden-object picture task or the transcription task. Graphs show the average over the marked channels (black circles) in the topographies. Shaded regions mark the standard error of the mean. Asterisks indicate the level of significance (^*^p ≤ 0.05; ^**^p ≤ 0.01; ^***^p ≤ 0.001). **(a)** Street conditions. Target sounds under the hidden-object picture task (HOP) and the transcription task. **(b)** Street conditions. Non-target sounds under the hidden-object picture task (HOP) and the transcription task.

We investigated the effect of the additional task under passive listening, where we hypothesized to find smaller N100 amplitudes in the street soundscape condition while working on the transcription task (block 3), which is closer to everyday life, than while working on the more experimental hidden object picture task (block 2). We tested this for bell and bird sounds respectively (Figure 6). Wilcoxon signed-rank test revealed a statistically significant difference between the bird sounds while working on the hidden-object picture task and the transcription task (adj. *p* ≤ 0.001, Z = –4.031, adjusted for 6 comparisons, Figure 6a), where the amplitude on the N100 was significantly larger while working on the hidden-object picture task (M = 0.1852 μV, SD = 0.760 μV) than while working on the transcription task (M = 0.769 μV, SD = 0.920 μV). We found no statistically significant difference for the bell sounds (adj. *p* = 0.050, Z = –1.958, adjusted for 6 comparisons, Figure 6b) in the expected direction, where the bell sound while working on the hidden-object picture task had a larger N100 amplitude (M = –0.371 μV, SD = 0.874 μV) than while working on the transcription task (M = 0.029 μV, SD = 0.410 μV). We therefore found evidence in favor of Hypothesis 4 concerning the bird sounds but not the bell sounds.

#### 3.3.5 Learning and unlearning of auditory relevance—hypothesis 5

**Figure 7:**
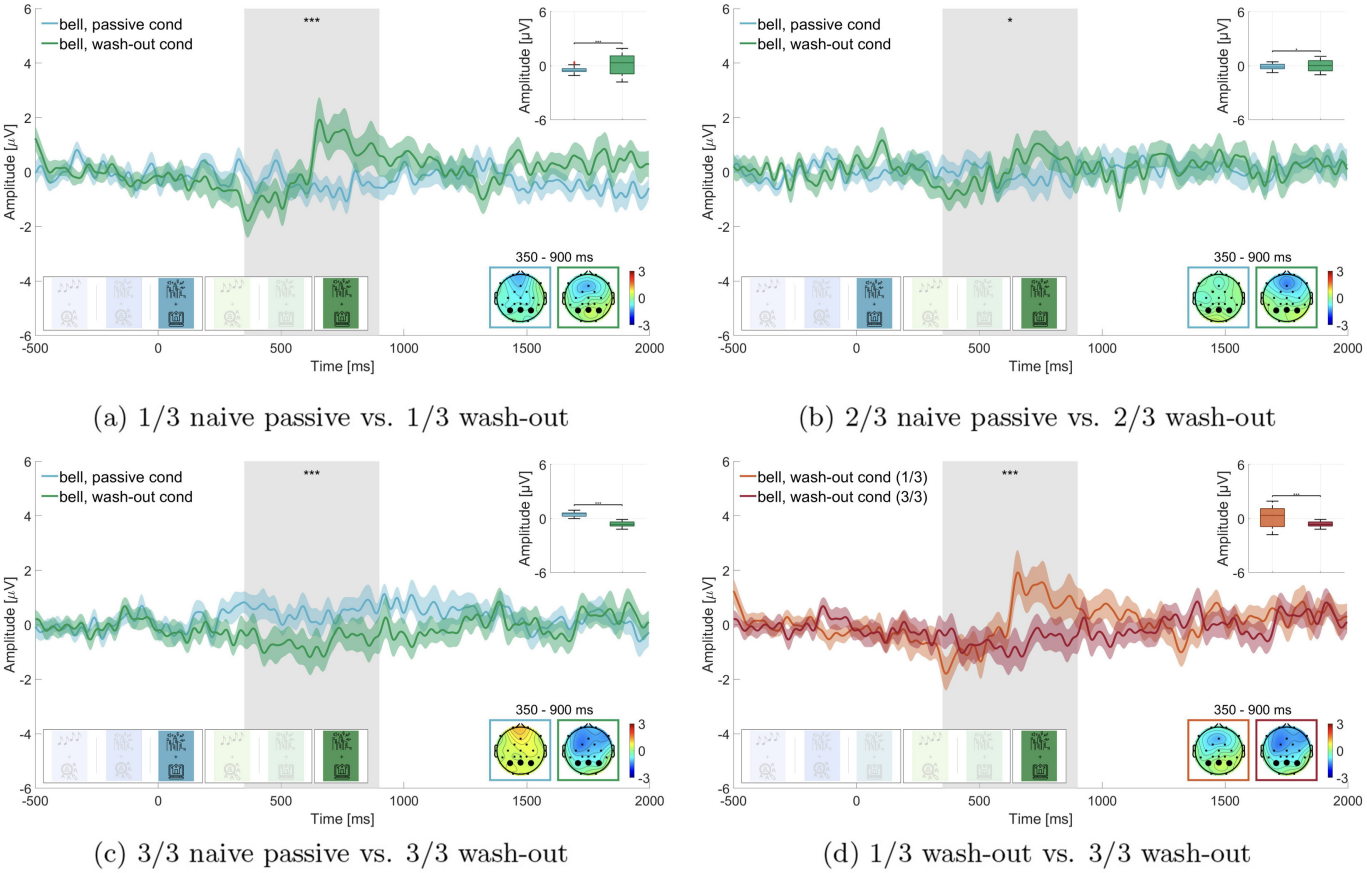
Comparison of the P300 amplitude in the passive naive listening condition vs. wash-out condition. Graphs show the average over the marked channels (black circles) in the topographies. Shaded regions mark the standard error of the mean. Asterisks indicate the level of significance (^*^p ≤ 0.05; ^**^p ≤ 0.01; ^***^p ≤ 0.001). **(a)** Street conditions. First third of all bell sounds while working on the transcription task in naive passive listening vs. wash-out. **(b)** Street conditions. Second third of all bell sounds while working on the transcription task in naive passive listening vs. wash-out. **(c)** Street conditions. Third third of all bell sounds while working on the transcription task in naive passive listening vs. wash-out. **(d)** Street conditions. First third of all bell sounds while working on the transcription task in wash-out vs. third third of all bell sounds while working on the transcription task in wash-out.

We tested whether we could effectively down-modulate the relevance of the bell in the wash-out block (block 6). We split the data into thirds and compared the P300 amplitude of the respective thirds of passive naive listening (block 3) and the wash-out block (block 6), as can be seen in Figure 7. We found that in the first third of the data the P300 amplitude of the wash-out block (M = 0.145 μV, SD = 1.094 μV) was significantly larger than in the passive naive listening block (M = –0.450 μV, SD = 0.317 μV; adj. *p* ≤ 0.01 Z = –4.851, adjusted for 6 comparisons, Figure 7a). In the second third the P300 amplitude of the wash-out block (M = 0.057 μV, SD = 0.616 μV) was not significantly different from the passive naive listening block (M = –0.108 μV, SD = 0.310 μV; adj. *p* = 0.022, Z = –2.288, adjusted for 6 comparisons, Figure 7b). For the final third, we found that, contrary to our expectation, the P300 amplitude in the passive naive listening condition was larger (M = 0.456 μV, SD = 0.243 μV) than in the wash-out condition (M = –0.631 μV, SD = 0.284 μV; adj. *p* ≤ 0.01, Z = 10.192, adjusted for 6 comparisons, Figure 7c).

To further investigate the wash-out effect, we also compared in the wash-out block the first third of trials to the last third of trials (Figure 7d) and found that the P300 amplitude was significantly larger in the first third of trials (M = 0.145 μV, SD = 1.094 μV) than in the final third of trials (M = –0.631 μV, SD = 0.284 μV; adj. *p* ≤ 0.01, Z = 6.672, adjusted for 4 comparisons).

## 4 Discussion

This study is part of a larger research project aimed at understanding the neural basis of sound perception in everyday life. Through a structured experimental design, we examined how to analyze EEG data in response to complex soundscapes within an office-like environment. By systematically increasing the complexity of the acoustic scene and the non-auditory task, our approach allows us to gain insights into how EEG data can be used to study (changes in) auditory processing in more ecologically valid conditions and over the course of more than 3 hours. This stepwise approach is essential for bridging the gap between the extensive body of lab-based research and the growing interest in understanding brain function in real-world scenarios.

### 4.1 Same sound, different response—how context forms our perception

Our study investigated the influence of contextual factors, particularly personal relevance, task complexity and stimuli properties, on sound perception. The results highlight how the properties of the non-auditory task significantly impact neural activity, specifically the N100 and P300 components. These findings emphasize that sound perception is shaped not only by the physical characteristics of stimuli but also by the context in which they are encountered, including the cognitive demands placed on the listener (Rimskaya-Korsakova et al., 2022; Debnath and Wetzel, 2022; Asutay and Västfjäll, 2012; Siegel and Stefanucci, 2011; Rosenkranz et al., 2023; Schlossmacher et al., 2021; Shinn-Cunningham and Best, 2008). This reinforces the idea that personal relevance and task complexity are critical factors in shaping sound perception, as introduced in our initial research questions.

These findings are consistent with previous work conducted in mobile and semi-naturalistic EEG settings. For instance, Ladouce et al. (2019) and Hölle and Bleichner (2023) demonstrated that it is feasible to track attention-related neural markers over extended periods and in ecologically valid conditions. Our study builds upon this by examining ERP components in response to experimentally manipulated auditory relevance and task complexity over 3.5 h, providing a novel perspective on how these markers behave in semi-structured yet dynamic environments. Furthermore, the context-dependent modulation of early auditory responses observed in our study aligns with the findings of Straetmans et al. (2021) and Gramann et al. (2014), who reported variability in neural responses linked to task and environmental constraints. We add to this work by showing that even within a stationary indoor setting, subtle differences in task complexity (e.g., transcription vs. visual search) can significantly shape ERP amplitudes.

#### 4.1.1 Stability of the P300 component

##### 4.1.1.1 Attention modulation of target processing—hypothesis 1

Our results demonstrate that the P300 component is relatively stable across different auditory scenes (Figure 3). The P300 exhibited a similar morphology in both the roving oddball and street soundscape conditions, suggesting that it is a robust measure of attention that is less influenced by the properties of the stimuli or the nature of the non-auditory task, which is in line with other research on the stability of the P300 component (Fallgatter et al., 2000). This finding reinforces the idea that the P300 can reliably indicate attentional processes in various contexts, making it a valuable tool in both laboratory and real-world settings (Polich, 2007). This stability across both isolated sounds and complex soundscapes suggests that the P300 as a marker of attention is a reliable measure across different auditory contexts, addressing our second research question. This result aligns with previous real-world EEG findings showing robust attentional markers in complex environments (Straetmans et al., 2021). However, unlike studies such as Ladouce et al. (2019), where attention shifts were tracked in freely moving participants, we maintained higher experimental control and focused on longer continuous EEG data with intermittent auditory relevance.

##### 4.1.1.2 Contextual influence on the P300 component—hypothesis 3

Contrary to our expectations, we observed a larger amplitude difference in the P300 window between passive and active listening for the street soundscape compared to the roving oddball (Figure 5). Although we hypothesized that the roving oddball would show a larger amplitude difference, the opposite was true. This unexpected finding could be attributed to the fact that the bell did not elicit a response within the designated time frame in the passive listening condition but a larger response in the active listening condition (cf. Figure 3b), leading to a larger difference in the P300 amplitude. With regard to the roving oddball, a slight potential was observed at the outset of the window of interest for the target sounds in the passive listening condition and a larger response in the active listening condition (cf. Figure 3a). This resulted in a smaller difference between passive and active listening, leading to the difference wave for the roving oddball being smaller than the difference wave for the street soundscape condition. As a limitation, the difference curve in the P300 window might not have aligned with our expectations due to the significant disparity in the number of trials analyzed. There were a maximum of 50 bell sounds in the street soundscape and up to 120 first tones in the roving oddball. The smaller number of trials in the street soundscape condition may have resulted in a larger neural response since the bell sound occurred only sporadically, as compared to the first tones in the roving oddball (Fitzgerald and Picton, 1984). Lastly, the low amount of trials in the street soundscape condition might have led to less reliable data, contributing to the unexpected outcomes in the difference wave analysis.

#### 4.1.2 Influence of task complexity on the N100 component

##### 4.1.2.1 Attention modulation of non-target processing—hypothesis 2

In contrast to the P300, the N100 component appears to be more sensitive to the properties of the stimuli and the non-auditory task. While the N100 response was stable for the non-relevant last beep tones in the roving oddball condition, we did not observe a comparable N100 response to the non-relevant bird calls in the street soundscape (Figure 4). While we refer to bird calls and final tones as “irrelevant,” we acknowledge that in the active listening condition, participants needed to monitor the full auditory scene in order to detect target sounds. Therefore, the observed N100 modulation may reflect general auditory attention demands rather than a strict distinction between attended and ignored stimuli. Interestingly, there was an N100-like component in the passive listening condition, but this component was absent in the active listening condition. This discrepancy might indicate cognitive resource allocation, as proposed in the attentional resources hypothesis (Huang and Elhilali, 2020), where resources in the active condition were potentially used up by the transcription task and the detection of the behaviorally relevant bells.

##### 4.1.2.2 Task complexity effects on early auditory processing (N100)–hypothesis 4

We found that the non-auditory task had a strong effect on the N100 response to bell sounds (Figure 6), which further supports the considerations on cognitive resource allocation. The N100 was present only during the simpler hidden-object picture task and was completely absent during the more complex transcription task. This suggests that the more demanding task may have consumed the cognitive resources necessary for processing of the irrelevant bell sounds. The observed variability in the N100 response further indicates that complex soundscapes, especially when combined with more demanding tasks, lead to more context-dependent neural responses compared to isolated sounds.

The N100 was present only during the simpler hidden-object picture task and was completely absent during the more complex transcription task. This suggests that the more demanding task may have consumed the cognitive resources necessary for processing of the irrelevant bell sounds. The observed variability in the N100 response further indicates that complex soundscapes, especially when combined with more demanding tasks, lead to more context-dependent neural responses compared to isolated sounds.

This supports previous findings that early auditory processing can be modulated by cognitive load and task requirements (Debnath and Wetzel, 2022; Schlossmacher et al., 2021). In contrast to Rosenkranz et al. (2023), who investigated attention in active multisensory tasks, our results provide evidence for attentional modulation of auditory ERP components even in the absence of overt attention tasks. This strengthens the argument that neural processing of sound is continuously influenced by task complexity, adding nuance to theories of auditory filtering in naturalistic environments (Shinn-Cunningham and Best, 2008).

We can rule out the possibility that the bell was simply not perceivable, as the same soundscape was played under both non-auditory tasks. Interestingly, the N100 response to the bird calls was observed under both tasks, although the amplitude was greater during the simpler task. This difference between the responses to the bell and bird calls might be due to the higher salience of the bird calls, which may have captured bottom-up attentional resources even when resources were otherwise allocated to the more complex task. An alternative explanation is that the hidden-object picture task always preceded the transcription task, which might have affected participants' neural responsiveness, leading to a reduced N100 in the transcription task due to prior exposure or task-related fatigue. However, this explanation is not supported by the behavioral data of the non-auditory task, as there are no significant differences in performance that would indicate exhaustion of the participants. It is unlikely that the absence of an N100 component for the bell sounds while working on the transcription task is a mere order effect, since it is not seen for the bird sounds. As a limitation to our study design, we did not include a questionnaire on the awareness of the additional sounds in the street soundscape which in hindsight would have provided further validation of our findings through the use of an additional behavioral measure.

Overall, our findings demonstrate the complex interaction of task complexity and sound characteristics. A defining characteristic of natural sound is that it does not inherently possess a sharp sound onset. This is particularly crucial when examining stimuli that are not inherently salient and are embedded within the context of ambient sound. Ambient sound can result in the energetic masking of target stimuli, which in turn affects their perceptual onset (Weise et al., 2012; Oganian and Chang, 2019). This may result in temporal smearing of the ERP, given that the perceptual onset is highly individual. Thus, when using more natural stimuli, researchers should be aware of the individual perceptual differences which may influence the effect. While more individualized analyses pipelines could be employed, e.g., by using individual time-windows to compute amplitude averages, our data showed a homogeneous perceptual onset. The high degree of coherence between participants is visible in the morphology of the ERP, where peaks are narrow.

#### 4.1.3 Learning and unlearning personal relevance

##### 4.1.3.1 Learning and unlearning of auditory relevance—hypothesis 5

Our study underlines, that relevance of sound can be learned and subsequently unlearned, independently of its physical properties. This is apparent from the gradual decrease in the P300 amplitude during the wash-out block (Figure 7). Our findings with complex stimulus material in a simulated office environment are in line with previous studies (Polich, 2007) and add on the opposite effect, where relevant stimuli can be unlearned again. This suggests that relevance of sound can effectively be unlearned. Nevertheless, it remains unclear whether this is a consequence of the passage of time or an effect of the number of repetitions of the former target sounds. In order to investigate this point further, these two alternative explanations should be tested independently of each other in a future study.

Moreover, it would be intriguing to ascertain whether noise-sensitive individuals demonstrate a comparable pattern of unlearning relevance to non-noise-sensitive individuals in a comparative study. If noise-sensitive individuals exhibit greater difficulty in unlearning the relevance of a specific sound, this could serve as a potential avenue for developing interventions to address noise sensitivity and would extend the findings on a potentially impaired gating mechanism in noise-sensitive individuals (Shepherd et al., 2016, 2019).

However, contrary to our expectations, the P300 amplitude in the final third of trials during the wash-out block was significantly lower than in the passive listening condition. While this could indicate an effect of intentional forgetting (Ten Oever et al., 2021), it is more likely due to random data drift, given the small number of trials analyzed in the street soundscape condition after splitting into thirds. We had to balance two competing factors: maintaining a naturalistic soundscape and ensuring enough trials for analysis. The choice to include 50 target sounds over 45 min helped preserve the natural feel of working next to an open window in the city, but it may have compromised data quality, particularly for the split-into-thirds analysis.

### 4.2 Bridging laboratory and real-world studies

Our study bridges the gap between controlled laboratory experiments and the unpredictable nature of real-world environments. By examining neural processing in a setting that mimics real-world conditions, we sought to understand how sound processing changes under different levels of experimental control. The results show that sound processing is influenced by multiple factors, including the complexity of the task, personal relevance, the properties of the sounds, and the broader environmental context. These findings suggest that certain neural markers, like the P300, generalize well from controlled lab environments to more complex, real-world-like scenarios. However, other components, such as the N100, exhibit greater sensitivity to task demands and may be less easily generalizable, thereby addressing our third research question.

One limitation of our study is that the order of the passive naive listening blocks was fixed rather than randomized. While we do not expect that this has influenced our results, randomizing these blocks could have reduced potential effects of participant fatigue across the experiment. Furthermore, the order of the active listening blocks was deliberately fixed to maintain the integrity of the wash-out phase. If these blocks had been randomized, some participants would have had a longer break from the street soundscape before starting the wash-out condition, which could have impacted the effect of relevance fading. By ensuring a consistent transition between these blocks, we aimed to preserve the mental representation of the bell sound. However, we acknowledge that maintaining a fixed order may have contributed to participant fatigue, particularly in the later blocks, which could have influenced neural responses. Future studies may consider alternative designs, such as counterbalancing passive blocks or implementing structured breaks to mitigate potential fatigue effects.

We found that even small changes in the task setup, such as whether participants' hands rested on the response key or had to move to it, led to significant differences in the recorded signal. We observed fewer artifacts in the hidden-object picture task, where there was a higher degree of experimental control, introduced by the fact that participants could keep their hand on the response key. In contrast, during the transcription task, which required more free movement of the hands and head (as participants looked down to locate keys), a substantial time-locked artifact emerged in the EEG data. These findings underscore how minor variations in task setup, particularly related to motor activity, can greatly impact data quality and should be carefully considered when interpreting results in less controlled environments (Gramann et al., 2014; Gramann, 2024; Jacobsen et al., 2021).

This highlights a general challenge when working with data in more naturalistic settings. While there are valid reasons to maintain consistent pre-processing across conditions, as we did following the protocol by Klug and Gramann (2021), this approach may not be ideal when artifact structures differ significantly between tasks. A limitation that arises from our setup is the relatively low number of EEG channels (24), which may reduce the spatial resolution of ICA-based artifact removal. While higher-density EEG systems can offer improved source separation, our use of a low-density setup reflects a deliberate trade-off: our goal was to design a system suitable for more naturalistic, real-life environments. On average, we removed 8 out of 24 components per participant (min = 4, max = 11), and interpolated 1.4 channels (min = 0, max = 5). Although this may impact the precision of artifact correction, we found that data quality remained sufficient for ERP analysis, supporting the feasibility of ecologically valid EEG research. In our study, appending the data from all conditions before applying ICA ensured uniform pre-processing and enabled cross-block comparisons. However, this approach may have limited the removal of task-specific artifacts, which might have been better addressed with condition-specific pre-processing pipelines.

A final point of consideration is the relatively high standard deviation in hit and false alarm rates in the roving oddball task. As highlighted in our behavioral analysis, this variability was largely driven by a small subset of participants whose performance deviated substantially from the group. When these outliers were excluded using a conservative IQR-based criterion, both the false alarm rate and hit rate stabilized considerably, reflecting more consistent performance across the remaining sample. This suggests that, while the task was generally well-performed, it may have posed challenges for a few individuals, possibly due to differences in attentional strategies or fatigue. These insights emphasize the importance of complementary behavioral analyses when interpreting neural data, especially in longer or cognitively demanding experiments.

### 4.3 Implications for future research

Our study builds on and extends earlier work in naturalistic EEG research. In doing so, it demonstrates the feasibility of obtaining valuable insights from EEG data in realistic conditions, aligning with previous work in this area (Ladouce et al., 2019; Jacobsen et al., 2021; Gramann et al., 2014; Reiser et al., 2021; Scanlon et al., 2022; Zink et al., 2016; Straetmans et al., 2021; Rosenkranz et al., 2024). Importantly, we showed that meaningful brain signals can be recorded over extended periods, even in the absence of specific instructions related to the sound stimuli. In support of this, we observed that signal quality could be maintained over the course of the full 3.5-h session. Impedance checks between blocks revealed only minimal need for intervention: re-gelling was required for fewer than half of the participants, mostly involving a single electrode, and no full cap reapplication was necessary.

This builds on the work of Hölle and Bleichner (2023), who demonstrated long-term EEG recording feasibility using ear-EEG. Our setup maintained higher signal quality and temporal resolution with a full-cap configuration, enabling ERP analyses that complement the envelope-based tracking methods commonly used in mobile EEG studies (Jaeger et al., 2020; Fuglsang et al., 2017). In doing so, we demonstrate that temporally precise event-related analyses are possible in long-duration, ecologically valid settings.

The ability to capture sound processing without active participant engagement is particularly promising for fields such as psychiatry, where it may help in studying impaired sensory gating in conditions like borderline personality disorder and schizophrenia, or in patients who may not comply with momentary assessment. The capacity to record EEG over extended periods in naturalistic settings, as demonstrated in our study, is pivotal for elucidating the neural mechanisms underlying the processing of complex auditory environments in everyday life. Importantly, our findings also underscore the necessity of preserving data integrity, as minor behavioral and environmental alterations can introduce unanticipated variability.

In light of these insights, future research should further explore sound perception in ecologically valid settings, while meticulously controlling for variables such as task complexity, environmental context, and personal relevance. Rather than a comprehensive transition to real-world experimentation, which introduces numerous uncontrolled variables, we propose a stepwise approach. This method permits researchers to incrementally introduce greater complexity while conducting validation checks between experimental blocks, thereby ensuring that observed neural changes are attributable to experimental manipulations rather than uncontrolled factors.

Specifically, future studies should examine how elements such as the acoustic properties of stimuli, task demands, and personal relevance shape neural processing. A more profound comprehension of these elements will empower researchers to devise experiments that capture real-world auditory complexity while maintaining scientific rigor.

The broader implications of our findings extend to other fields of beyond-the-lab research. As the number of studies investigating cognitive and neural processes in naturalistic environments increases, it is becoming increasingly evident that minor variations in task or environmental conditions can have a considerable impact on neural responses. This highlights the importance of meticulous experimental design and careful consideration of the context in which data is collected. By adopting a controlled, stepwise approach, researchers can systematically address the challenges of studying auditory perception in naturalistic environments, such as the variability introduced by task demands and environmental factors, which we highlighted in the introduction.

### 4.4 Conclusion: insights on sound perception

In our study, we explored three key questions related to sound perception in naturalistic environments: (1) how personal relevance affects the perception of soundscapes over time, (2) how neural responses differ between isolated sounds and complex soundscapes, and (3) whether lab-based findings generalize to more naturalistic settings.

Our results suggest that sound perception is highly context-dependent, with task complexity and personal relevance playing significant roles in shaping neural responses, as mirrored by the N100 and P300 component. We demonstrated that the P300 is relatively stable across different auditory conditions, making it a reliable marker of attention even in dynamic environments. However, the N100 showed greater variability, suggesting that it is more sensitive to the complexity of the task and properties of the stimuli. Our findings further emphasize that personal relevance plays a critical role in determining how individuals process auditory stimuli in complex environments, further supporting the idea that sound perception is not only a function of the physical properties of sound but also its relevance to the listener.

We also found that results from controlled lab environments may not fully generalize to real-world settings, as task demands and environmental factors can significantly alter neural responses. However, by adopting a middle-ground approach, our study provides insights into how EEG can be used to study sound perception in more ecologically valid environments without sacrificing the precision of lab-based studies. Future research should continue to explore these questions, refining experimental designs to better capture the complexities of real-world auditory processing.

## Data Availability

The datasets presented in this study can be found in online repositories. The names of the repository/repositories and accession number(s) can be found below: https://zenodo.org/records/14011457.

## Footnotes

^1^https://www.youtube.com/watch?v=Le_g4s6KloU, (accessed July 1, 2022).

^2^https://www.youtube.com/watch?v=uya2CXwCY5w, (accessed July 1, 2022).

^3^for further information see: https://www.zooniverse.org/projects/courtaulddigital/world-architecture-unlocked/about/research.

^4^https://github.com/labstreaminglayer/liblsl-Matlab, v1.14.0.

^5^https://github.com/labstreaminglayer/App-Input, v1.15.0.

^6^https://github.com/labstreaminglayer/App-LabRecorder, v1.14.0.
