# Supplementary material for: From beeps to streets: unveiling sensory input and relevance across auditory contexts
Source: Front Neuroergon. 2025 Apr 28;6:1571356. doi: 10.3389/fnrgo.2025.1571356 (PMC12066603; doi:10.3389/fnrgo.2025.1571356)
Supplement: Supplementary file 1 [file Data_Sheet_1.pdf]

10 Supplementary Material

10.1 Participant exclusion

Block	N	Reason for exclusion
1	22	1x bad data quality
2	22	1x bad data quality
3	22	1x bad data quality
4	21	1x missing data,<br>1x bad data quality
5	21	1x bad performance in auditory task,<br>1x bad data quality and non-compliance in non-auditory task
6	21	1x bad performance in auditory task in former block,<br>1x bad data quality and non-compliance in non-auditory task

Table 1: Overview of reasons for excluding participants from the ERP and statistical analyses per block

10.2 Rationale for the multiple comparison correction

The following corrections were applied in each hypothesis:

- Hypothesis 1 involved two comparisons: First tones epochs block 1 vs. first tones epochs block 4 and bells epochs block 3 vs. bells epochs block 5. Since the bells epochs from block 3 were used in 6 comparisons across Hypotheses 1, 3, 4, and 5, we applied a Bonferroni correction for 6 comparisons to all tests within Hypothesis 1. This ensures that the repeated use of bells epochs from block 3 across multiple hypotheses is properly accounted for.

- Hypothesis 2 involved two comparisons: last tones epochs block 1 vs. last tones epochs block 4 and birds epochs block 3 vs. birds epochs block 5. Here, the birds epochs from block 3 were used in only 2 comparisons across Hypotheses 2 and 4. Therefore, a Bonferroni correction for 2 comparisons was applied to all tests within hypothesis 2.

- Hypothesis 3 involved one comparison: first tones epochs block 4 minus first tones epochs block 1 vs. bells epochs block 5 minus bells epochs block 3. Given that the bells epochs from block 3 were used in 6 comparisons across multiple hypotheses, the correction for this hypothesis was also based on 6 comparisons.

- Hypothesis 4 included two comparisons: bells epochs block 2 vs. bells epochs block 3 and birds epochs block 2 vs. birds epochs block 3. Since bells epochs from block 3 were used in 6 comparisons and birds epochs from block 3 in 2 comparisons across multiple hypotheses, we applied a Bonferroni correction for 6 comparisons, ensuring consistency within the hypothesis while accounting for the maximum number of relevant tests.

- Hypothesis 5 involved four comparisons between thirds of bell epochs from block 3 and Block 6. As bell epochs from block 3 were used in 6 comparisons across hypotheses, a Bonferroni correction for 6 comparisons was applied to all tests within Hypothesis 5.
